# Self-Reported Screen Time on Social Networking Sites Associated With Problematic Smartphone Use in Chinese Adults: A Population-Based Study

**DOI:** 10.3389/fpsyt.2020.614061

**Published:** 2021-01-14

**Authors:** Ningyuan Guo, Tzu Tsun Luk, Man Ping Wang, Sai Yin Ho, Daniel Yee Tak Fong, Alice Wan, Sophia Siu-chee Chan, Tai Hing Lam

**Affiliations:** ^1^School of Nursing, University of Hong Kong, Hong Kong, China; ^2^School of Public Health, University of Hong Kong, Hong Kong, China; ^3^Aberdeen Kai-fong Welfare Association Social Service, Hong Kong, China

**Keywords:** screen time, screen-based activities, problematic smartphone use, addictive symptoms, social networking sites

## Abstract

**Background:** Problematic smartphone use (PSU) has been associated with screen time in general, but little is known about the effect of different screen-based activities. We examined the associations of self-reported time spent on overall and specific screen-based activities with PSU and its addictive symptoms in Hong Kong Chinese adults.

**Methods:** We analyzed data from 562 smartphone owners (56.5% female; 82.1% aged 25–64 years) in a population-based telephone survey in 2017. PSU was measured using Smartphone Addiction Scale-Short Version (range 10–60) which includes symptoms of daily-life disturbance, withdrawal, cyberspace-oriented relationship, overuse, and tolerance. Screen time was self-reported as average hours per day spent on the internet, online book/newspaper/magazine, online video, and social networking sites (SNS). Multivariable linear regression analyzed the associations of self-reported screen time with PSU severity and symptoms. Interaction effects of sex, age group, educational attainment, and monthly household income were examined.

**Results:** Self-reported time spent on overall screen-based activities was associated with PSU severity (β = 1.35, 95% CI 0.15, 2.55) and withdrawal and overuse symptoms, after adjusting for sociodemographic and health-related variables. Independent association was observed for self-reported SNS time with PSU severity (β = 1.42, 95% CI 0.35, 2.49) and symptoms of withdrawal and cyberspace-oriented relationship, after mutually adjusting for time on other activities. The strongest association between self-reported SNS time and PSU severity was observed in younger than older adults (β = 4.36, 95% CI 2.58, 6.13; *P* for interaction = 0.004).

**Conclusions:** The independent association of self-reported SNS time with PSU and core addictive symptoms highlighted the addiction potential of SNS use, particularly in younger users.

## Introduction

Excessive use of the internet has raised concerns about its behavioral addiction potential (i.e., internet use disorders), among which online gaming disorder has been added in ICD-11 (1). Problematic smartphone use (PSU) is suggested as a subtype of internet use disorder (2) showing the similar neurobiological pathway of structural and functional brain abnormalities with addictive behaviors (e.g., gambling disorder) (3, 4). PSU could represent symptoms of salience, mood modification, withdrawal, tolerance, conflict, and relapse as posited in the behavioral addiction components model (5, 6). Such symptoms have a spectrum of severity that can be less or more significant to indicate pathological addiction (7). Core criteria of PSU were proposed to include symptoms related to withdrawal, tolerance, and functional impairments (of physical/psychological health, social relationship, or workplace/school performance) (7, 8). The diagnostic accuracy of the criteria has been shown in studies to distinguish online gaming disorder from non-pathological use (9). However, it should be noted that PSU is not an official diagnosis in ICD-11 or DSM-5 and may not be comparable to the established heroin and tobacco addictions on severity and/or associated health problems (7). Debates are ongoing on whether PSU qualifies for an addiction (10, 11). We used the term “PSU” as recommended to avoid over-pathologizing of everyday-life behaviors (12–14).

PSU is found to be associated with self-reported overall screen time (15, 16). The association was supported in studies objectively measuring smartphone use on different devices (Android and iOS) (17–19). The screen time can involve various activities differing in using motives categorized into process and social use (20–23). Process use is for news consumption, entertainment, or other nonsocial motives, such as surfing the internet/websites, reading online book/newspaper/magazine, and watching online videos, while social use is for social interaction motives, such as using social networking sites (SNS; e.g., Facebook, Twitter, WhatsApp, WeChat) (21).

Results are mixed on associations between different screen-based activities and PSU. Social-oriented activity was associated with PSU (15), particularly in females who might have greater sociality than males and younger people who might be more active on SNS (24). Exposure to social comparison information on SNS, such as a large amount of “Likes” and comments, perfect body images, and idealized lives, might reduce self-esteem and increase depression (25), which are consistently associated with PSU (26–28). Other research showed stronger associations of process-oriented activities with PSU adjusting for sex and age (20). Spending more time on process-oriented activities could be a mechanism for individuals with higher anxiety to deflect negative stressors (29), while anxiety and dysfunctional emotion regulation could lead to PSU (20). Little is known about the potential moderating role of educational attainment and income, despite that electronic literacy and pattern of engagement in screen-based activities may differ by socioeconomic status (30).

We used the Uses and Gratifications Theory (UGT) (31) and the Interaction of Person-Affect-Cognition-Execution (I-PACE) model for addictive behaviors (32) to theoretically conceptualize the study. The UGT posits that individual differences motivate people to increasingly use specific types of electronic media to satisfy underlying needs (31). UGT was proposed for traditional media but has been widely applied to the advanced screen-based activities (20, 21, 26). Individual differences in the UGT can include sociodemographic and psychological characteristics such as anxiety and depression symptoms (21). The more comprehensive I-PACE model can be used to explain associations between specific screen-based activities and PSU (23, 33). Specific using motives plus general characteristics (e.g., sociodemographic and psychological characteristics) could predispose individuals to the onset and maintenance of PSU as posited in the I-PACE model (32).

Hong Kong, the most developed city in China, has one of the highest smartphone penetration rates worldwide (91.5% in 2019) (34). Our previous study showed a high prevalence of PSU (38.5%) in Hong Kong (35). We have also found that PSU was associated with impaired mental health and family well-being (36, 37). This study took advantage of a population-based survey in Hong Kong Chinese adults to examine the associations of time spent on overall and specific types of screen-based activities with PSU and its addictive symptoms. We also examined the potential interaction effects of sex, age group, educational attainment, and monthly household income on these associations.

## Materials and Methods

### Design and Participants

The Hong Kong Family and Health Information Trends Survey (FHInTS) was a periodic territory-wide telephone survey on the general public's behaviors and views regarding information use, individual health, and family well-being, under the project “FAMILY: A Jockey Club Initiative for a Harmonious Society.” We conducted five waves of FHInTS since 2009. The present landline telephone survey was part of the fifth wave of FHInTS from February to August 2017. Details of the study design have been reported elsewhere (30, 35). The target population was Cantonese-speaking Hong Kong residents aged 18 years or above. A two-stage probability-based sampling method was used to minimalize sampling bias. In the first stage of random-digit-dialing, telephone numbers were randomly generated using known prefixes assigned to telecommunication service providers by the Government Office of the Communications Authority, which covered nearly all households in Hong Kong. Invalid numbers were removed according to the computer and manual dialing records. Telephone numbers of respondents from previous waves were also filtered. In the second stage of within household sampling, once a household was successfully reached, an eligible family member whose next birthday was the closest to the interview day was invited for the survey. All telephone interviews were conducted by trained interviewers from Public Opinion Programme (POP) at the University of Hong Kong. Of 5,773 eligible subjects, 4,054 responded to the survey (response rate = 70.2%). Six hundred eighty-six respondents were randomly selected to answer questions on screen time and PSU. The final study sample comprised 562 smartphone owners, after excluding smartphone nonowners.

### Measures

PSU was measured using the ten-item Smartphone Addiction Scale-Short Version (SAS-SV), with each item scoring on a six-point Likert scale (1 = strongly disagree to 6 = strongly agree) (38). A higher total SAS-SV score (range 10–60) indicates a higher PSU severity (38). The addictive symptoms of cyberspace-oriented relationship, overuse, and tolerance each have one item; symptoms of daily-life disturbance have three items; and withdrawal symptoms have four items (38). The average score of these multi-item symptoms was calculated (range 1–6). The Chinese version of SAS-SV was found reliable and valid in our previous study (35). Cronbach's alpha was 0.84 in the present sample. SAS-SV scores could be dichotomized into “non-PSU” and “PSU” using suggested cutoffs (male 31; female 33) (38). However, the cutoffs were established by receiver-operating characteristic analyses on adolescents (38), which may be less applicable to adults in the present study. Note that PSU has not been an official diagnosis but a behavior ranging from unproblematic to problematic (7). We hence used continuous SAS-SV scores for all analyses.

Respondents were asked that “In the past month, how many hours did you spend on…per day? [If <1 h, please probe how long, i.e., half an hour (0.5), 15 min (0.25), no (0)].” Process-oriented screen-based activities included surfing the internet; reading online book/newspaper/magazine; watching online video (e.g., YouTube); and social-oriented activity included using SNS (e.g., Facebook, Twitter, WhatsApp, WeChat). We categorized the amount of time as “0,” “>0 to <1 hours per day,” “≥1 to <2 hours per day,” “≥2 to <3 hours per day,” and “≥3 hours per day.” The similar categorization was used in large-scale longitudinal studies on self-reported screen time (25, 39). Overall screen time was calculated by summing the amount of time spent on all four screen-based activities measured.

Sociodemographic characteristics included sex, age group, marital status, employment status, educational attainment (primary or below, secondary, or tertiary), and monthly household income (≤HK$ 19,999, 20,000–29,999, or ≥30,000; US $1 = HK $7.8) (the median household income was HK$ 24,900 in Hong Kong in 2016). Cigarette smoking (never smoker, former smoker, or current smoker) and alcohol drinking (never drinker, former drinker, occasional drinker, or monthly or more drinker) were examined, given the co-occurrence of substance use and addictive behaviors (40). History of doctor-diagnosed chronic diseases (e.g., cardiovascular diseases, respiratory diseases, liver diseases, allergies, and others) was dichotomized into none or any. Psychological characteristics were measured using the validated four-item Patient Health Questionnaire (PHQ-4) (41). Each item scores on a Likert scale (0 = not at all to 3 = nearly every day), with a higher total score (range 0–12) indicating higher symptom severity (41). Cronbach's α was 0.83 in the present sample. Cigarette smoking, alcohol drinking, chronic disease, and PHQ-4 score were grouped as health-related characteristics.

### Statistical Analysis

All data were weighted according to sex, age, and educational attainment distributions of the Hong Kong general population to increase the sample representativeness. We examined the associations of time spent on overall and each of four specific screen-based activities with PSU severity and symptoms using bivariate and multivariable linear regression analyses adjusting for sociodemographic and health-related characteristics (Model 1). In Model 2, we repeated Model 1 with mutually adjusting time spent on other types of screen-based activities. The variance inflation factors (range 1.51–2.34, <10 acceptable) suggested the minimal multicollinearity among time spent on different types of screen-based activities in Model 2 (42). Dichotomized SAS-SV scores by suggested cutoffs were used for testing robustness of results in sensitivity analyses. We further tested whether any observed association of time spent on specific screen-based activity with PSU severity in Model 2 differed by sex, age group, educational attainment, and monthly household income by adding multiplicative interaction terms. An omnibus *P* for interactor was calculated using adjusted Wald tests. Missing data were handled by available case analyses as missing values for all variables were minimal (<2.0%). All analyses were conducted using STATA version/MP 15.1 (StataCorp., TX, USA). A two-side *P* < 0.05 was considered statistically significant.

## Results

The weighted sample (*N* = 562; 56.5% female; 82.1% aged 25–64 years) had a mean SAS-SV score of 27.8 [standard deviation (SD) 10.2], with the highest symptom score was observed for cyberspace-oriented relationship (mean 3.5, SD 1.6) (Table 1).

**Table 1:** Demographic characteristics and SAS-SV total and symptom score (*N* = 562).

	**Nonweighted (*N* = 497)**	**Weighted^a^ (*N* = 562)**
	***n* (%)**	***n* (%)**
**Sex**		
Male	192 (38.6)	244 (43.5)
Female	305 (61.4)	318 (56.5)
**Age group, years**		
18–24	67 (13.5)	54 (9.6)
25–44	98 (19.8)	248 (44.1)
45–64	216 (43.5)	214 (38.0)
≥65	116 (23.3)	47 (8.3)
**Marital status**		
Unmarried	141 (28.4)	205 (36.5)
Cohabitated/married	303 (61.0)	318 (56.6)
Divorced/separated/widowed	53 (10.7)	39 (6.9)
**Employment status**		
Unemployed	20 (4.0)	32 (5.7)
In-paid employed	202 (40.6)	315 (56.0)
Retired	143 (28.8)	77 (13.6)
Housekeeper	85 (17.1)	98 (17.4)
Full-time student	47 (9.5)	41 (7.3)
**Educational attainment**		
Primary or below	61 (12.3)	90 (16.0)
Secondary	222 (44.7)	279 (49.6)
Tertiary	214 (43.1)	193 (34.4)
**Monthly household income (HK $)^b^**		
≤9,999	70 (14.1)	44 (8.0)
10,000–19,999	60 (12.1)	79 (14.1)
20,000–29,999	95 (19.1)	124 (22.0)
30,000–39,999	63 (12.7)	90 (16.1)
≥40,000	143 (28.8)	158 (28.1)
Unstable/refused to answer	66 (13.3)	67 (11.9)
**Cigarette smoking**		
Never smoker	409 (82.3)	438 (77.9)
		
Former smoker	51 (10.3)	68 (12.0)
Current smoker	37 (7.4)	57 (10.1)
**Alcohol drinking**		
Never drinker	220 (44.3)	228 (40.6)
Former drinker	19 (3.8)	23 (4.1)
Occasional drinker	181 (36.4)	217 (38.6)
Monthly or more drinker	77 (15.5)	94 (16.7)
**Chronic disease diagnosis**		
None	324 (65.2)	420 (74.7)
Any	173 (34.8)	142 (25.3)
**PHQ-4 score, range 0–12, mean ± SD**	2.0 ± 2.4	2.3 ± 2.7
**SAS-SV score, range 10–60, mean ± SD**	26.8 ± 9.7	27.8 ± 10.2
**SAS-SV symptom score, range 1–6, mean ± SD**		
Daily-life disturbance^c^	2.3 ± 1.1	2.5 ± 1.2
Withdrawal^d^	3.0 ± 1.3	3.0 ± 1.3
Cyberspace-oriented relationship	3.5 ± 1.6	3.5 ± 1.6
Overuse	2.6 ± 1.4	2.8 ± 1.5
Tolerance	1.9 ± 1.2	2.0 ± 1.3

*PHQ-4, 4-item Patient Health Questionnaire; SAS-SV, Smartphone Addiction Scale-Short Version; SD, standard deviation*.

*Reported by n (%), otherwise as indicated*.

a *Weighted by sex, age, and educational attainment distributions of Hong Kong general population*.

b *US $1 = HK $7.8*.

c *The average score of the symptoms of daily-life disturbance that contains three items*.

d *The average score of withdrawal symptoms that contains four items*.

Nearly three-quarters of respondents (71.5%) reported spending over 3 h per day on overall screen-based activities (Table 2). The most prevalent activity (spent over 3 h per day) was surfing the internet, followed by using SNS, reading online book/newspaper/magazine, and watching online video.

**Table 2:** Hours per day spent on overall and specific types of screen-based activities (*N* = 562).

**Time spent on screen-based activities, hours/day**	**Nonweighted (*N* = 497)**	**Weighted^a^ (*N* = 562)**
	***n* (%)**	***n* (%)**
**Overall**		
0	15 (3.1)	10 (1.8)
>0 to <1	46 (9.4)	38 (6.9)
≥1 to <2	63 (12.9)	51 (9.2)
≥2 to <3	51 (10.4)	59 (10.6)
≥3	314 (64.2)	397 (71.5)
**Surfing the internet**		
0	90 (18.1)	71 (12.7)
>0 to <1	66 (13.3)	63 (11.3)
≥1 to <2	94 (19.0)	109 (19.5)
≥2 to <3	74 (15.0)	80 (14.3)
≥3	171 (34.6)	237 (42.2)
**Reading online book/newspaper/magazine**		
0	204 (41.1)	211 (37.5)
>0 to <1	111 (22.4)	130 (23.1)
≥1 to <2	110 (22.2)	123 (21.9)
≥2 to <3	42 (8.5)	51 (9.1)
≥3	29 (5.9)	47 (8.4)
**Watching online video**		
0	219 (44.2)	214 (38.2)
>0 to <1	109 (22.0)	137 (24.5)
≥1 to <2	90 (18.2)	117 (21.0)
≥2 to <3	41 (8.3)	47 (8.4)
≥3	36 (7.3)	44 (7.9)
**Using social networking sites**		
0	35 (7.1)	26 (4.7)
>0 to <1	147 (29.9)	151 (27.1)
≥1 to <2	136 (27.6)	137 (24.6)
≥2 to <3	62 (12.6)	94 (16.9)
≥3	112 (22.8)	150 (26.8)

a *Weighted by sex, age, and educational attainment distributions of Hong Kong general population*.

Multivariable analyses showed that each hour of increase in time spent on overall screen-based activities was associated with higher symptom severity of withdrawal (adjusted β = 0.19, 95% CI 0.05, 0.34) and overuse (adjusted β = 0.20, 95% CI 0.02, 0.38) and higher PSU severity (adjusted β = 1.35, 95% CI 0.15, 2.55), after adjusting for sociodemographic and health-related characteristics (Model 1) (Table 3). Associations with higher PSU severity were also observed for each hour of increase in time spent on surfing the internet, reading online book/newspaper/magazine, watching online video, and using SNS in Model 1. After mutually adjusting for time spent on other types of screen-based activities (Model 2), these associations were attenuated and became nonsignificant except for time spent on using SNS. Each hour of increase in time spent on using SNS was associated with higher PSU severity (adjusted β = 1.42, 95% CI 0.35, 2.49) and higher symptom severity of withdrawal (adjusted β = 0.18, 95% CI 0.05, 0.32) and cyberspace-oriented relationship (adjusted β = 0.38, 95% CI 0.23, 0.53). The association of SNS time with PSU was found robust in sensitivity analyses (adjusted odds ratio = 1.40, 95% CI 1.08, 1.83) (Supplementary Table 1). Each hour of increase in time spent on surfing the internet was associated with higher symptom severity of daily-life disturbance (adjusted β = 0.13, 95% CI 0.02, 0.24). Each hour of increase in time spent on watching online video was associated with higher symptom severity of withdrawal (adjusted β = 0.13, 95% CI 0.004, 0.26).

**Table 3:** Associations of time spent on overall and specific screen-based activities with PSU and its addictive symptoms (*N* = 562).

**Time spent on screen-based activities**	**Regression model**	**Association with SAS-SV score, β (95% CI)**	**Association with SAS-SV symptom score**, **β (95% CI)**				
			**Daily-life disturbance**	**Withdrawal**	**Cyberspace-oriented relationships**	**Overuse**	**Tolerance**
Overall	Crude	1.23 (0.18, 2.29)^*^	0.12 (-0.01, 0.25)	0.11 (−0.03, 0.24)	0.08 (−0.08, 0.23)	0.27 (0.11, 0.43)^**^	0.06 (−0.09, 0.20)
	Model 1^a^	1.35 (0.15, 2.55)^*^	0.06 (−0.08, 0.20)	0.19 (0.05, 0.34)^**^	0.15 (−0.04, 0.34)	0.20 (0.02, 0.38)^*^	0.07 (−0.10, 0.23)
Surfing the internet	Crude	1.51 (0.67, 2.36)^***^	0.19 (0.10, 0.28)^***^	0.11 (−0.001, 0.22)	0.06 (−0.07, 0.18)	0.29 (0.17, 0.41)^***^	0.11 (0.005, 0.22)^*^
	Model 1^a^	1.76 (0.77, 2.76)^**^	0.16 (0.05, 0.27)^**^	0.19 (0.07, 0.32)^**^	0.12 (−0.02, 0.27)	0.23 (0.09, 0.38)^**^	0.14 (0.01, 0.28)^*^
	Model 2^b^	0.89 (−0.14, 1.91)	0.13 (0.02, 0.24)^*^	0.08 (−0.06, 0.22)	−0.03 (−0.19, 0.12)	0.12 (−0.04, 0.28)	0.08 (−0.07, 0.22)
Reading online book/newspaper/magazine	Crude	1.37 (0.37, 2.36)^**^	0.13 (0.02, 0.24)^*^	0.11 (−0.01, 0.24)	0.09 (−0.06, 0.23)	0.29 (0.16, 0.42)^***^	0.11 (−0.02, 0.23)
	Model 1^a^	1.55 (0.60, 2.49)^**^	0.10 (−0.01, 0.21)	0.17 (0.06, 0.28)^**^	0.17 (0.03, 0.32)^*^	0.24 (0.10, 0.38)^**^	0.14 (0.01, 0.27)^*^
	Model 2^b^	0.68 (−0.24, 1.60)	0.03 (−0.08, 0.13)	0.07 (−0.05, 0.19)	0.09 (−0.06, 0.24)	0.14 (−0.001, 0.28)	0.07 (−0.06, 0.20)
Watching online video	Crude	1.53 (0.58, 2.47)^**^	0.17 (0.05, 0.28)^**^	0.15 (0.02, 0.27)^*^	0.07 (−0.08, 0.23)	0.24 (0.10, 0.38)^**^	0.10 (−0.03, 0.22)
	Model 1^a^	1.39 (0.45, 2.34)^**^	0.07 (−0.04, 0.19)	0.20 (0.09, 0.31)^**^	0.10 (−0.05, 0.26)	0.15 (0.01, 0.29)^*^	0.10 (−0.05, 0.26)
	Model 2^b^	0.77 (−0.29, 1.83)	0.03 (−0.09, 0.15)	0.13 (0.004, 0.26)^*^	0.02 (−0.15, 0.17)	0.06 (−0.09, 0.21)	0.05 (−0.11, 0.21)
Using social networking sites	Crude	1.60 (0.70, 2.50)^**^	0.13 (0.02, 0.23)^*^	0.14 (0.02, 0.26)^*^	0.24 (0.10, 0.38)^**^	0.29 (0.16, 0.42)^***^	0.11 (−0.01, 0.23)
	Model 1^a^	1.99 (0.94, 3.05)^***^	0.10 (−0.02, 0.22)	0.24 (0.12, 0.36)^***^	0.38 (0.24, 0.51)^***^	0.24 (0.09, 0.40)^**^	0.14 (0.002, 0.28)^*^
	Model 2^b^	1.42 (0.35, 2.49)^**^	0.04 (−0.07, 0.15)	0.18 (0.05, 0.32)^**^	0.38 (0.23, 0.53)^***^	0.16 (−0.002, 0.32)	0.10 (−0.04, 0.24)

*PSU, problematic smartphone use; SAS-SV, Smartphone Addiction Scale-Short Version*.

*All data were weighted by sex, age, and educational attainment distribution of Hong Kong general population*.

* *P < 0.05*,

** *P < 0.01*,

*** *P < 0.001*.

a *Adjusted for sex, age, marital status, educational attainment, employment status, monthly household income, cigarette smoking, alcohol drinking, chronic disease, and four-item Patient Health Questionnaire score*.

b *Additionally adjusted for time spent on other screen-based activities*.

We also examined the interaction effects of sex, age group, educational attainment, and monthly household income on the association of SNS time with PSU severity in Model 2 (Table 4). Younger respondents aged 18–24 years had the strongest association (adjusted β = 4.36, 95% CI 2.58, 6.13), compared with older respondents (25–44 years: adjusted β = 0.97, 95% CI −0.76, 2.69; 45–64 years: adjusted β = 1.69, 95% CI 0.35, 3.04; ≥65 years: adjusted β = 2.87, 95% CI 0.95, 4.80; *P* for interaction = 0.004). Sex appeared to have no interaction effect on the association of SNS time with PSU severity (male: adjusted β = 1.52, 95% CI 0.03, 3.02; female: adjusted β = 1.98, 95% CI 0.46, 3.51). Respondents with higher educational attainment tended to have the stronger association of SNS time with PSU severity (secondary: adjusted β = 1.46, 95% CI 0.11, 2.82; tertiary: adjusted β = 2.11, 95% CI 0.90, 3.33), but the interaction effect did not reach significance level (*P* for interaction = 0.06).

**Table 4:** Adjusted associations of time spent on using social networking sites with PSU by sex, age, educational attainment, and monthly household income (*N* = 562)^a^.

	**SAS-SV score, mean ± SD**	**Adjusted associations of social networking sites time with SAS-SV score**	
		**β (95% CI)**	***P* for interaction**
**Sex**			0.17
Male	28.0 ± 9.7	1.52 (0.03, 3.02)^*^	
Female	27.7 ± 10.5	1.98 (0.46, 3.51)^*^	
**Age group, years**			0.004
18–24	29.5 ± 8.6	4.36 (2.58, 6.13)^***^	
25–44	28.3 ± 6.8	0.97 (−0.76, 2.69)	
45–64	27.1 ± 11.6	1.69 (0.35, 3.04)^*^	
≥65	26.8 ± 16.9	2.87 (0.95, 4.80)^**^	
**Educational attainment**			0.06
Primary or below	30.5 ± 9.9	0.13 (−3.37, 3.63)	
Secondary	26.5 ± 9.5	1.46 (0.11, 2.82)^*^	
Tertiary	28.4 ± 10.6	2.11 (0.90, 3.33)^**^	
**Monthly household income (HK $)^b^**			0.06
≤19,999	28.0 ± 12.9	0.90 (−0.61, 2.42)	
20,000–29,999	27.9 ± 8.6	2.54 (0.41, 4.68)^*^	
≥30,000	27.6 ± 9.8	0.85 (−0.74, 2.44)	

*PSU, problematic smartphone use; SAS-SV, Smartphone Addiction Scale-Short Version*.

*All data were weighted by sex, age, and educational attainment distribution of Hong Kong general population*.

* *P < 0.05*,

** *P < 0.01*,

*** *P < 0.001*.

a *Adjusted for sex, age, marital status, educational attainment, employment status, monthly household income, cigarette smoking, alcohol drinking, chronic disease, four-item Patient Health Questionnaire score, and time spent on other screen-based activities*.

b *US $1 = HK $7.8*.

## Discussion

In a random sample of Hong Kong Chinese adults, self-reported time spent on overall screen-based activities was positively associated with PSU severity. This finding was consistent with studies showing that longer smartphone usage time was associated with higher PSU severity using the same SAS-SV in Swiss, Spanish, and Belgian populations (15, 16).

Our study added to the literature by exploring the associations of self-reported time spent on overall and specific types of screen-based activities with PSU and its addictive symptom. Specifically, we found that overall screen time was associated with overuse (i.e., “Using my smartphone longer than I had intended.”). The self-awareness of excessive use was also reported in a qualitative interview in users with higher PSU severity (8). However, overuse has been identified as a necessary but insufficient condition of pathological addiction (43). Tolerance symptoms were proposed in the core criteria (7), but we observed no association of overall screen time with tolerance. This might be attributable to the failure of the single-item measure of tolerance in SAS-SV (i.e., “The people around me tell me that I use my smartphone too much.”) to capture other aspects, including increases in financial costs, feelings of gratification, and achievement (e.g., game score/level and SNS “Like”/comment) (44). Overall screen time was also not associated with core addictive symptoms of daily-life disturbance or cyberspace-oriented relationship. In contrast, impairments of workplace/school performance and offline interactions were consistently observed in research into online gaming disorder, a specific form of internet use disorder (9).

Self-reported time spent on using SNS was independently associated with PSU and its core addictive symptoms, including withdrawal and cyberspace-oriented relationship. This association was observed after adjusting for time spent on reading online book/newspaper/magazine, watching online video, and surfing the internet. The findings showed that social- and process-oriented activity were not equally associated with PSU, which supported the UGT (31) and I-PACE (32). Some SNS has features to predispose users to excessive use to meet such higher levels of online social demands; Snapchat is an example that delivers messages only available for a short time after having been viewed by recipients (45). The ephemeral nature of Snapchat can risk users to PSU to avoid missing out the time-limited social connections (46). Our observed association of SNS time with withdrawal symptoms complemented the findings from an experimental study, which found that imagining no access to SNS for 48 h led to dysregulated emotions, depression, and stress (47). A potential mediator on this association might be social anxiety about missing out real-time posts, events, and interactions on SNS (48). The observed association of SNS time with cyberspace-oriented relationship suggested a trade-off between online and offline interactions in the displacement hypothesis (49), which was also supported by impaired family relationships associated with increased SNS time (50). The independent associations of self-reported SNS time with PSU highlighted the needs of specific measurements on problematic SNS use (51) and interventions to prevent and reduce SNS time.

Younger adults appeared to be the most susceptible to the association of self-reported SNS time with PSU in our subgroup analyses. A similar finding was reported in a cross-generation study showing the predictive effect of SNS time on PSU only in the younger group (52). Younger adults tend to be more active but have lower self-control than older adults in using SNS, which might explain the observed association (24). Early adulthood is a developmental stage of emotion change, self-control underdevelopment, and reward sensitivity, which are known risk factors for PSU (6). Neuroscience studies supported the notion by identifying the lateral orbitofrontal gray matter abnormalities in young people with PSU and particularly in those spent time on SNS, and the lateral orbitofrontal cortex is important in regulatory control and reward-related decision-making (3). The stronger association of SNS time with PSU was also observed in those with higher education in our study, although the interaction effect was not significant possibly due to insufficient sample size. Higher SES group may have more social capitals, but greater needs to develop and maintain social connections have been associated with increased SNS time (53).

Self-reported time spent on surfing the internet was associated with daily-life disturbance in our study. Internet has an array of contents for information seeking, shopping, gaming, pornography viewing, gambling, or aimless browsing, and some of which have been identified with addiction potential (e.g., gaming and gambling disorders) and were associated with impairments of school/work performance (54, 55). We found that time spent on watching online video was associated with withdrawal symptoms, which was consistent with a study showing anxiety symptoms associated with excessive use of process-oriented activity (20). The popular video-sharing and live-streaming SNS (e.g., TikTok, Twitch, and Facebook Live) suggested that social anxiety about missing out connections may also be a mediator in the pathway from online video time to withdrawal symptoms (56). Future studies would benefit from examining more detailed contents that are used on the internet and online video.

Our results have theoretical and practical implications. The different associations between time spent on social- and process-oriented activities and PSU informed future research to distinguish between using motives. This fits with the UGT (31) and I-PACE (32) which posit specific motives could predispose individuals to PSU. Prevention and intervention programs, particularly in younger adults, might target limiting SNS time to reduce PSU severity and symptoms. Technology providers could consider incorporate time limit setting into mobile devices or applications.

Our study has several limitations. The cross-sectional data restricted the inference of the temporal sequence of self-reported screen time and PSU. Reverse direction of the association is possible as people with PSU tend to have longer screen time to deflect negative emotions induced by PSU (57). Longitudinal, experimental, and intervention studies are warranted to clarify causal relations and potential mechanisms. Although we adjusted for several potential confounders consistently reported in the literature (24), residual or unmeasured confounders such as personalities of low self-control and social anxiety might explain the observed associations between screen time and PSU (6, 48). Landline survey excluding mobile phone-only group might not be representative for the entire population. Weighting can reduce the non-coverage bias but may not fully compensate for differences in screen time and PSU between landline and mobile surveys. The exclusion of individuals younger than 18 years might underestimate the findings as this age group tended to have longer screen time and higher risks for PSU (24). Self-reported screen time was subject to recall bias and social desirability bias and with uncertain reliability. Future research can use the reliable screen-time questionnaire (58) or objective smartphone use by longitudinal and repeated measures (17–19) to validate the results. The lacking data on other popular social- (e.g., email) and process-oriented activities (e.g., gaming, shopping, online banking) warranted more comprehensive measures in future research.

## Conclusions

Self-reported screen time was positively associated with PSU severity in Chinese adults in Hong Kong, but the associations varied by types of screen-based activities. The independent associations of self-reported SNS time with PSU severity and core addictive symptoms of withdrawal and cyberspace-oriented relationship highlighted recent warnings about excessive SNS use. Younger adults were the most susceptible to the association of SNS time with PSU severity. Our study provided reminders to smartphone users of the addiction potential of screen time in particular on using SNS. The study could help SNS providers and policymakers to develop regulations to prevent excessive SNS use and PSU.

## Data Availability Statement

The data analyzed in this study is subject to the following licenses/restrictions: The data that support the findings of this study are available from the FAMILY project but restrictions apply to the availability of these data, which were used under license for the current study, and so are not publicly available. Data are however available from the authors upon reasonable request and with permission of the Hong Kong Jockey Club Charities Trust. Requests to access these datasets should be directed to FAMILY project, https://www.family.org.hk/en/about-us/contact-us-en/.

## Ethics Statement

The studies involving human participants were reviewed and approved by the Institutional Review Board of the University of Hong Kong/Hospital Authority Hong Kong West Cluster. All respondents provided verbal informed consent. Telephone interviews were audio-recorded for quality checking with respondents' consent. Interview records were then erased 6 months after completing the survey. Written informed consent for participation was not required for this study in accordance with the national legislation and the institutional requirements.

## Author Contributions

NG: formal analysis, data curation, writing—original draft, and writing—review and editing. TTL: writing—review and editing. MPW: conceptualization, methodology, and writing—review and editing, supervision. SYH: conceptualization, methodology, writing—review and editing, and supervision. DYTF: writing—review and editing and supervision. AW: project administration. SSC: conceptualization and methodology. THL: conceptualization, methodology, writing—review and editing, and funding acquisition. All authors contributed to the article and approved the submitted version.

## Conflict of Interest

The authors declare that the research was conducted in the absence of any commercial or financial relationships that could be construed as a potential conflict of interest.
