# Supplementary material for: Self-Reported Screen Time on Social Networking Sites Associated With Problematic Smartphone Use in Chinese Adults: A Population-Based Study
Source: Front Psychiatry. 2021 Jan 14;11:614061. doi: 10.3389/fpsyt.2020.614061 (PMC7840886; doi:10.3389/fpsyt.2020.614061)
Supplement: Supplementary file 1 [file Table_1.DOCX]

Supplementary Table 1. Associations of time spent on overall and specific screen-based activities with PSU (N=562)

| Time spent on screen-based activities | Regression model | Association with dichotomized SAS-SV score, odds ratio (95% CI) |
| --- | --- | --- |
|  |  |  |
| Overall | Crude | 1.20 (0.92, 1.56) |
|  | Model 1 ^a^ | 1.22 (0.90, 1.63) |
| Surfing the internet | Crude | 1.18 (0.98, 1.42) |
|  | Model 1 ^a^ | 1.21 (0.96, 1.52) |
|  | Model 2 ^b^ | 1.12 (0.86, 1.45) |
| Reading online book/newspaper  /magazine | Crude | 1.04 (0.84, 1.29) |
|  | Model 1 ^a^ | 1.08 (0.86, 1.34) |
|  | Model 2 ^b^ | 0.94 (0.73, 1.21) |
| Watching online video | Crude | 1.16 (0.95, 1.42) |
|  | Model 1 ^a^ | 1.10 (0.87, 1.40) |
|  | Model 2 ^b^ | 1.01 (0.78, 1.30) |
| Using social networking sites | Crude | 1.23 (1.00, 1.51)* |
|  | Model 1 ^a^ | 1.40 (1.08, 1.82)* |
|  | Model 2 ^b^ | 1.40 (1.08, 1.83)* |

PSU, problematic smartphone use; SAS-SV, Smartphone Addiction Scale-Short Version.

SAS-SV scores were dichotomized into PSU (32.2%) and non-PSU (67.8%) by cut-offs (male 31; female 33).

All data were weighted by sex, age, and educational attainment distribution of Hong Kong general population.

**P*<0.05, ***P*<0.01, ****P*<0.001.

^a^ Adjusted for sex, age, marital status, educational attainment, employment status, monthly household income, cigarette smoking, alcohol drinking, chronic disease, and 4-item Patient Health Questionnaire score.

^b^ Additionally adjusted for time spent on other screen-based activities.
