# Supplementary material for: Self-Reported Screen Time on Social Networking Sites Associated With Problematic Smartphone Use in Chinese Adults: A Population-Based Study
Source: Front Psychiatry. 2021 Jan 14;11:614061. doi: 10.3389/fpsyt.2020.614061 (PMC7840886; doi:10.3389/fpsyt.2020.614061)
Supplement: Supplementary file 2 [file Data_Sheet_1.PDF]

```
svyset [pweight=demoweight]
/* Table 1 data preparation */
*sex
ge demo_sex=DM01
label values demo_sex DM01
*age group
recode DM03gp1 (1=1 "18-24") (2/3=2 "25-44") (4/5=3 "45-64") (6=4 "65+"), ge (demo_age)
*marital status
recode  DM06 (1=1 "unmarried") (2/3=2 "cohabitated/married") (4/6=3 "divorced/separated/separated"), ge
(demo_maritalstatus)
*Education attainment
ge demo_edu = DM07gp_TP
label values demo_edu DM07gp_TP
*Employment status
fre DM05
recode DM05 (4/5=1 "unemployment") (9=1 "unemployment") (1/3=2 "in paid employment") (8=3 "retired") (6=4
"housekeeper") (7=5 "fulltime student") , ge (demo_employment)
*Monthly household income (HK$)
recode  DM08ax (1/3=1 "<=9999") (4=2 "10000-19999")(5=3 "20000-29999") (6=4 "30000-39999") (7/9=5
"40000+") (8886=0 "unstable/refused") (-99=0 "unstable/refused" ) , ge (dome_income)
*Cigarette smoking
recode AT04x (1/2=1 "current") (3=2 "former" ) (4=3 "never") , ge (sh_smoking)
*Alcohol drinking
recode AT01a (-99=.) (8886 = 0 "never") (8885 =1 "former") (8884=2 "occasional") ( 1=2 "occasional") (2/5 =3
"monthly or more")  , ge (sh_drinking)
*Chronic disease
recode SH11x_8886 (8886=0 "no") (0=1 "yes") , ge (sh_cd)
*Psychological characteristcis
recode SH04_1 (-99=.) (1=0 "never") (2=1 "less than 7 days") (3=2 "7 days or more") (4=3 "nearly daily"), ge
(gad1)
recode SH04_2 (-99=.) (1=0 "never") (2=1 "less than 7 days") (3=2 "7 days or more") (4=3 "nearly daily"), ge
(gad2)
recode SH04_3 (-99=.) (1=0 "never") (2=1 "less than 7 days") (3=2 "7 days or more") (4=3 "nearly daily"), ge
(phq1)
recode SH04_4 (-99=.) (1=0 "never") (2=1 "less than 7 days") (3=2 "7 days or more") (4=3 "nearly daily"), ge
(phq2)
ge sh_phq = gad1+ gad2 + phq1 + phq2
alpha gad1 gad2 phq1 phq2 if  DU01x_2==2&Type==1
*Smartphone addiction scale-short version
recode DU14_1(-99=.) (1=1) (2=2) (3=3) (4=4) (5=5)(6=6) , ge (sas1)
recode DU14_2(-99=.) (1=1) (2=2) (3=3) (4=4) (5=5)(6=6) , ge (sas2)
recode DU14_3(-99=.) (1=1) (2=2) (3=3) (4=4) (5=5)(6=6) , ge (sas3)
recode DU14_4(-99=.) (1=1) (2=2) (3=3) (4=4) (5=5)(6=6) , ge (sas4)
recode DU14_5(-99=.) (1=1) (2=2) (3=3) (4=4) (5=5)(6=6) , ge (sas5)
recode DU14_6(-99=.) (1=1) (2=2) (3=3) (4=4) (5=5)(6=6) , ge (sas6)
recode DU14_7(-99=.) (1=1) (2=2) (3=3) (4=4) (5=5)(6=6) , ge (sas7)
recode DU14_8(-99=.) (1=1) (2=2) (3=3) (4=4) (5=5)(6=6) , ge (sas8)
recode DU14_9(-99=.) (1=1) (2=2) (3=3) (4=4) (5=5)(6=6) , ge (sas9)
recode DU14_10(-99=.) (1=1) (2=2) (3=3) (4=4) (5=5)(6=6) , ge (sas10)
ge sas= sas1+ sas2 +sas3 +sas4+ sas5+ sas6 +sas7 +sas8 +sas9 +sas10
ge lifedisturbance= sas1+ sas2+ sas3
ge lifedisturbance_sd=lifedisturbance/3
ge withdrawal=sas4+ sas5+ sas6 +sas7
ge withdrawa_sd=withdrawal/4
ge cyberrelation=sas8
ge overuse= sas9
ge tolerance = sas10
```

```
recode sas (min/30=0 "no" ) (31/max=1 "yes") if  demo_sex ==1 , ge (psu_male)
recode sas (min/32=0 "no" ) (33/max=1 "yes") if  demo_sex ==2 , ge (psu_female)
ge psu = psu_male
replace psu = psu_female if psu==.

/* Table 1 data presentation */
tab demo_sex if Type==1& DU01x_2 ==2
tab demo_age if Type==1& DU01x_2 ==2
tab demo_maritalstatus if Type==1& DU01x_2 ==2
tab demo_employment if Type==1& DU01x_2 ==2
tab demo_edu if Type==1& DU01x_2 ==2
tab demo_income if Type==1& DU01x_2==2
tab  sh_smoking if Type==1& DU01x_2 ==2
tab  sh_drinking if Type==1& DU01x_2 ==2
tab  sh_cd if Type==1& DU01x_2 ==2 //DU01x_2 ==2 means smartphone owner
sum sh_phq4 if Type==1& DU01x_2 ==2, d
sum sas if Type==1,d
sum lifedisturbance_sd if Type==1,d
sum withdrawal_sd if Type==1,d
sum cyberrelation if Type==1,d
sum overuse if Type==1,d
sum tolerance if Type==1,d
svy:tab demo_sex if Type==1& DU01x_2==2, col count
svy:tab demo_age if  Type==1& DU01x_2==2 ,col count
svy:tab demo_maritalstatus if Type==1& DU01x_2==2 ,col count
svy:tab demo_employment if  Type==1& DU01x_2==2 ,col count
svy:tab demo_edu if Type==1& DU01x_2==2 ,col count
svy:tab demo_income if Type==1& DU01x_2==2,col count
svy: tab  sh_smoking if Type==1& DU01x_2 ==2, count col
svy: tab  sh_drinking if Type==1& DU01x_2 ==2, count col
svy: tab  sh_cd if Type==1& DU01x_2 ==2, count col
svy: mean  sh_phq4 if Type==1& DU01x_2 ==2
estat sd
svy:mean sas if Type==1
estat sd
svy:mean lifedisturbance_sd if Type==1
estat sd
svy:mean withdrawal_sd if Type==1
estat sd
svy:mean cyberrelation if Type==1
estat sd
svy:mean overuse if Type==1
estat sd
svy:mean tolerance if Type==1
estat sd

/* Table 2 data preparation */
recode DU04x_4 (-99=.), ge (time_internet)
recode DU04x_5 (-99=.), ge (time_book)
recode DU04x_6 (-99=.), ge (time_video)
recode DU04x_7 (-99=.), ge (time_social media)
recode DU04x_8 (-99=.), ge (time_im)
ge time_sns = time_im + time_socialmedia
ge overalltime= time_internet+ time_book+ time_video+ time_sns
recode time_internet (0=0) (0.001/0.999=1 "0-1") (1/1.999=2 "1-2")(2/2.999=3 "2-3")(3/max=4 ">3"), ge
(time_internet_cati)
recode time_book (0=0) (0.001/0.999=1 "0-1") (1/1.999=2 "1-2")(2/2.999=3 "2-3")(3/max=4 ">3"), ge
```

```
(time_book_cati)
recode time_video (0=0) (0.001/0.999=1 "0-1") (1/1.999=2 "1-2")(2/2.999=3 "2-3")(3/max=4 ">3"), ge
(time_video_cati)
recode time_sns (0=0) (0.001/0.999=1 "0-1") (1/1.999=2 "1-2")(2/2.999=3 "2-3")(3/max=4 ">3"), ge ( time_sns_cati
)
recode overalltime (0=0) (0.001/0.999=1 "0-1") (1/1.999=2 "1-2")(2/2.999=3 "2-3")(3/max=4 ">3"), ge
(time_overall_cati)

/* Table 2 data presentation */
tab time_internet_cati if DU01x_2 ==2&Type==1
tab time_book_cati if DU01x_2 ==2&Type==1
tab time_video_cati if DU01x_2 ==2&Type==1
tab time_sns_cati if DU01x_2 ==2&Type==1
tab time_overall_cati if DU01x_2 ==2&Type==1
svy:tab time_internet_cati if  DU01x_2 ==2&Type==1,count col
svy:tab time_book_cati if  DU01x_2 ==2&Type==1,count col
svy:tab time_video_cati if  DU01x_2 ==2&Type==1,count col
svy:tab time_sns_cati if  DU01x_2 ==2&Type==1,count col
svy:tab time_overall_cati if DU01x_2 ==2&Type==1,count col

/* Table 3 */
* Crude model
svy:regress sas time_internet_cati
svy:regress lifedisturbance_sd time_internet_cati
svy:regress withdrawal_sd time_internet_cati
svy:regress cyberrelation time_internet_cati
svy:regress overuse time_internet_cati
svy:regress tolerance time_internet_cati

svy:regress sas time_book_cati
svy:regress lifedisturbance_sd time_book_cati
svy:regress withdrawal_sd time_book_cati
svy:regress cyberrelation time_book_cati
svy:regress overuse time_book_cati
svy:regress tolerance time_book_cati

svy:regress sas time_video_cati
svy:regress lifedisturbance_sd time_video_cati
svy:regress withdrawal_sd time_video_cati
svy:regress cyberrelation time_video_cati
svy:regress overuse time_video_cati
svy:regress tolerance time_video_cati

svy:regress sas time_sns_cati
svy:regress lifedisturbance_sd time_sns_cati
svy:regress withdrawal_sd time_sns_cati
svy:regress cyberrelation time_sns_cati
svy:regress overuse time_sns_cati
svy:regress tolerance time_sns_cati

svy:regress sas time_overall_cati
svy:regress lifedisturbance_sd time_overall_cati
svy:regress withdrawal_sd time_overall_cati
svy:regress cyberrelation time_overall_cati
svy:regress overuse time_overall_cati
svy:regress tolerance time_overall_cati
```

```
* Adjusted model 1
svy:regress sas time_internet_cati i.demo_age i.demo_sex i.demo_edu i.demo_income i.demo_maritalstatus
i.demo_employment i.sh_smoking i.sh_drinking i.sh_cd sh_phq4
svy:regress lifedisturbance_sd time_internet_cati i.demo_age i.demo_sex i.demo_edu i.demo_income
i.demo_maritalstatus i.demo_employment i.sh_smoking i.sh_drinking i.sh_cd sh_phq4
svy:regress withdrawal_sd time_internet_cati   i.demo_age i.demo_sex i.demo_edu i.demo_income
i.demo_maritalstatus i.demo_employment i.sh_smoking i.sh_drinking i.sh_cd sh_phq4
svy:regress cyberrelation time_internet_cati  i.demo_age i.demo_sex i.demo_edu i.demo_income
i.demo_maritalstatus i.demo_employment i.sh_smoking i.sh_drinking i.sh_cd sh_phq4
svy:regress overuse time_internet_cati   i.demo_age i.demo_sex i.demo_edu i.demo_income i.demo_maritalstatus
i.demo_employment i.sh_smoking i.sh_drinking i.sh_cd sh_phq4
svy:regress tolerance time_internet_cati  i.demo_age i.demo_sex i.demo_edu i.demo_income i.demo_maritalstatus
i.demo_employment i.sh_smoking i.sh_drinking i.sh_cd sh_phq4

svy:regress sas time_book_cati i.demo_age i.demo_sex i.demo_edu i.demo_income i.demo_maritalstatus
i.demo_employment  i.sh_smoking i.sh_drinking i.sh_cd sh_phq4
svy:regress lifedisturbance_sd time_book_cati i.demo_age i.demo_sex i.demo_edu i.demo_income
i.demo_maritalstatus i.demo_employment  i.sh_smoking i.sh_drinking i.sh_cd sh_phq4
svy:regress withdrawal_sd time_book_cati   i.demo_age i.demo_sex i.demo_edu i.demo_income
i.demo_maritalstatus i.demo_employment  i.sh_smoking i.sh_drinking i.sh_cd sh_phq4
svy:regress cyberrelation time_book_cati  i.demo_age i.demo_sex i.demo_edu i.demo_income i.demo_maritalstatus
i.demo_employment  i.sh_smoking i.sh_drinking i.sh_cd sh_phq4
svy:regress overuse time_book_cati   i.demo_age i.demo_sex i.demo_edu i.demo_income i.demo_maritalstatus
i.demo_employment  i.sh_smoking i.sh_drinking i.sh_cd sh_phq4
svy:regress tolerance time_book_cati  i.demo_age i.demo_sex i.demo_edu i.demo_income i.demo_maritalstatus
i.demo_employment  i.sh_smoking i.sh_drinking i.sh_cd sh_phq4

svy:regress sas time_video_cati i.demo_age i.demo_sex i.demo_edu i.demo_income i.demo_maritalstatus
i.demo_employment i.sh_smoking i.sh_drinking i.sh_cd sh_phq4
svy:regress lifedisturbance_sd time_video_cati i.demo_age i.demo_sex i.demo_edu i.demo_income
i.demo_maritalstatus i.demo_employment i.sh_smoking i.sh_drinking i.sh_cd sh_phq4
svy:regress withdrawal_sd time_video_cati   i.demo_age i.demo_sex i.demo_edu i.demo_income
i.demo_maritalstatus i.demo_employment i.sh_smoking i.sh_drinking i.sh_cd sh_phq4
svy:regress cyberrelation time_video_cati  i.demo_age i.demo_sex i.demo_edu i.demo_income i.demo_maritalstatus
i.demo_employment i.sh_smoking i.sh_drinking i.sh_cd sh_phq4
svy:regress overuse time_video_cati   i.demo_age i.demo_sex i.demo_edu i.demo_income i.demo_maritalstatus
i.demo_employment i.sh_smoking i.sh_drinking i.sh_cd sh_phq4
svy:regress tolerance time_video_cati  i.demo_age i.demo_sex i.demo_edu i.demo_income i.demo_maritalstatus
i.demo_employment i.sh_smoking i.sh_drinking i.sh_cd sh_phq4

svy:regress sas time_sns_cati i.demo_age i.demo_sex i.demo_edu i.demo_income i.demo_maritalstatus
i.demo_employment i.sh_smoking i.sh_drinking i.sh_cd sh_phq4
svy:regress lifedisturbance_sd time_sns_cati i.demo_age i.demo_sex i.demo_edu i.demo_income
i.demo_maritalstatus i.demo_employment i.sh_smoking i.sh_drinking i.sh_cd sh_phq4
svy:regress withdrawal_sd time_sns_cati   i.demo_age i.demo_sex i.demo_edu i.demo_income i.demo_maritalstatus
i.demo_employment i.sh_smoking i.sh_drinking i.sh_cd sh_phq4
svy:regress cyberrelation time_sns_cati  i.demo_age i.demo_sex i.demo_edu i.demo_income i.demo_maritalstatus
i.demo_employment i.sh_smoking i.sh_drinking i.sh_cd sh_phq4
svy:regress overuse time_sns_cati   i.demo_age i.demo_sex i.demo_edu i.demo_income i.demo_maritalstatus
i.demo_employment i.sh_smoking i.sh_drinking i.sh_cd sh_phq4
svy:regress tolerance time_sns_cati  i.demo_age i.demo_sex i.demo_edu i.demo_income i.demo_maritalstatus
i.demo_employment i.sh_smoking i.sh_drinking i.sh_cd sh_phq4

svy:regress sas time_overall_cati i.demo_age i.demo_sex i.demo_edu i.demo_income i.demo_maritalstatus
i.demo_employment  i.sh_smoking i.sh_drinking i.sh_cd sh_phq4
svy:regress lifedisturbance_sd time_overall_cati i.demo_age i.demo_sex i.demo_edu i.demo_income
i.demo_maritalstatus i.demo_employment  i.sh_smoking i.sh_drinking i.sh_cd sh_phq4
```

```
svy:regress withdrawal_sd time_overall_cati   i.demo_age i.demo_sex i.demo_edu i.demo_income
i.demo_maritalstatus i.demo_employment i.sh_smoking i.sh_drinking i.sh_cd sh_phq4
svy:regress cyberrelation time_overall_cati  i.demo_age i.demo_sex i.demo_edu i.demo_income
i.demo_maritalstatus i.demo_employment  i.sh_smoking i.sh_drinking i.sh_cd sh_phq4
svy:regress overuse time_overall_cati   i.demo_age i.demo_sex i.demo_edu i.demo_income i.demo_maritalstatus
i.demo_employment i.sh_smoking i.sh_drinking i.sh_cd sh_phq4
svy:regress tolerance time_overall_cati  i.demo_age i.demo_sex i.demo_edu i.demo_income i.demo_maritalstatus
i.demo_employment  i.sh_smoking i.sh_drinking i.sh_cd sh_phq4

* Adjusted model 2
svy:regress sas time_internet_cati time_book_cati time_video_cati time_sns_cati i.demo_age i.demo_sex
i.demo_edu i.demo_income i.demo_maritalstatus i.demo_employment  i.sh_smoking i.sh_drinking i.sh_cd sh_phq4
svy:regress lifedisturbance_sd time_internet_cati time_book_cati time_video_cati time_sns_cati i.demo_age
i.demo_sex i.demo_edu i.demo_income i.demo_maritalstatus i.demo_employment  i.sh_smoking i.sh_drinking
i.sh_cd sh_phq4
svy:regress withdrawal_sd time_internet_cati time_book_cati time_video_cati time_sns_cati  i.demo_age
i.demo_sex i.demo_edu i.demo_income i.demo_maritalstatus i.demo_employment  i.sh_smoking i.sh_drinking
i.sh_cd sh_phq4
svy:regress cyberrelation time_internet_cati time_book_cati time_video_cati time_sns_cati  i.demo_age i.demo_sex
i.demo_edu i.demo_income i.demo_maritalstatus i.demo_employment  i.sh_smoking i.sh_drinking i.sh_cd sh_phq4
svy:regress overuse time_internet_cati time_book_cati time_video_cati time_sns_cati  i.demo_age i.demo_sex
i.demo_edu i.demo_income i.demo_maritalstatus i.demo_employment   i.sh_smoking i.sh_drinking i.sh_cd sh_phq4
svy:regress tolerance time_internet_cati time_book_cati time_video_cati time_sns_cati i.demo_age i.demo_sex
i.demo_edu i.demo_income i.demo_maritalstatus i.demo_employment  i.sh_smoking i.sh_drinking i.sh_cd sh_phq4
svy:regress  time_internet_cati time_book_cati time_video_cati time_sns_cati
display "tolerance = " 1-e(r2) " VIF = " 1/(1-e(r2))
svy:regress  time_book_cati time_internet_cati  time_video_cati time_sns_cati
display "tolerance = " 1-e(r2) " VIF = " 1/(1-e(r2))
svy:regress  time_video_cati time_book_cati time_internet_cati   time_sns_cati
display "tolerance = " 1-e(r2) " VIF = " 1/(1-e(r2))
svy:regress time_sns_cati time_video_cati time_book_cati time_internet_cati
display "tolerance = " 1-e(r2) " VIF = " 1/(1-e(r2))

/* Sensitivity analyses using dichotomized SAS-SV scores */
* Crude model
svy:logistic psu  time_internet_cati
svy:logistic psu  time_book_cati
svy:logistic psu  time_video_cati
svy:logistic psu  time_sns_cati
svy:logistic psu  time_overall_cati

*Adjusted model 1
svy:logistic psu  time_internet_cati i.demo_age i.demo_sex i.demo_edu i.demo_income i.demo_maritalstatus
i.demo_employment  i.sh_smoking i.sh_drinking i.sh_cd sh_phq4
svy:logistic psu  time_book_cati i.demo_age i.demo_sex i.demo_edu i.demo_income i.demo_maritalstatus
i.demo_employment  i.sh_smoking i.sh_drinking i.sh_cd sh_phq4
svy:logistic psu  time_video_cati i.demo_age i.demo_sex i.demo_edu i.demo_income i.demo_maritalstatus
i.demo_employment  i.sh_smoking i.sh_drinking i.sh_cd sh_phq4
svy:logistic psu  time_sns_cati i.demo_age i.demo_sex i.demo_edu i.demo_income i.demo_maritalstatus
i.demo_employment  i.sh_smoking i.sh_drinking i.sh_cd sh_phq4
svy:logistic psu  time_overall_cati  i.demo_age i.demo_sex i.demo_edu i.demo_income i.demo_maritalstatus
i.demo_employment  i.sh_smoking i.sh_drinking i.sh_cd sh_phq4

*Adjusted model 2
svy:logistic psu  time_internet_cati time_book_cati time_video_cati time_sns_cati i.demo_age i.demo_sex
i.demo_edu i.demo_income i.demo_maritalstatus i.demo_employment  i.sh_smoking i.sh_drinking i.sh_cd sh_phq4
```

```
*table 4
recode demo_income (0=.) (1/2=1 "0-19999") (3=2 " 20000-29999") (4/5=3 ">30000") , ge (demo_income_cati)
svy:mean sas if Type==1, over (demo_sex)
estat sd
svy:mean sas if Type==1, over (demo_age)
estat sd
svy:mean sas if Type==1, over (demo_edu)
estat sd
svy:mean sas if Type==1, over (demo_income_cati)
estat sd

**SNS screen time
svy:regress sas i.time_sns_cati##i.demo_sex i.demo_age i.demo_edu i.demo_income i.demo_maritalstatus
i.demo_employment i.time_internet_cati i.time_book_cati i.time_video_cati i.sh_smoking i.sh_drinking i.sh_cd
sh_phq4
testparm i.time_sns_cati#i.demo_sex
svy:regress sas i.time_sns_cati##i.demo_age i.demo_sex  i.demo_edu i.demo_income i.demo_maritalstatus
i.demo_employment i.time_internet_cati i.time_book_cati i.time_video_cati i.sh_smoking i.sh_drinking i.sh_cd
sh_phq4
testparm i.time_sns_cati#i.demo_age
svy:regress sas i.time_sns_cati##i.demo_edu i.demo_age i.demo_sex   i.demo_income i.demo_maritalstatus
i.demo_employment i.time_internet_cati i.time_book_cati i.time_video_cati i.sh_smoking i.sh_drinking i.sh_cd
sh_phq4
testparm i.time_sns_cati#i.demo_edu
svy:regress sas i.time_sns_cati##i.demo_income_cati i.demo_maritalstatus i.demo_edu i.demo_age i.demo_sex
i.demo_employment i.time_internet_cati i.time_book_cati i.time_video_cati i.sh_smoking i.sh_drinking i.sh_cd
sh_phq4
testparm i.time_sns_cati#i.demo_income_cati

svy:regress sas time_sns_cati i.demo_edu i.demo_age i.demo_income i.demo_maritalstatus i.demo_employment
i.time_internet_cati i.time_book_cati i.time_video_cati i.sh_smoking i.sh_drinking i.sh_cd sh_phq4 if demo_sex==1
svy:regress sas time_sns_cati i.demo_edu i.demo_age i.demo_income i.demo_maritalstatus i.demo_employment
i.time_internet_cati i.time_book_cati i.time_video_cati i.sh_smoking i.sh_drinking i.sh_cd sh_phq4 if demo_sex==2

svy:regress sas time_sns_cati  i.demo_sex i.demo_edu   i.demo_income i.demo_maritalstatus i.demo_employment
i.time_internet_cati i.time_book_cati i.time_video_cati i.sh_smoking i.sh_drinking i.sh_cd sh_phq4 if demo_age==1
svy:regress sas time_sns_cati  i.demo_sex i.demo_edu   i.demo_income i.demo_maritalstatus i.demo_employment
i.time_internet_cati i.time_book_cati i.time_video_cati i.sh_smoking i.sh_drinking i.sh_cd sh_phq4  if
demo_age==2
svy:regress sas time_sns_cati  i.demo_sex i.demo_edu   i.demo_income i.demo_maritalstatus i.demo_employment
i.time_internet_cati i.time_book_cati i.time_video_cati i.sh_smoking i.sh_drinking i.sh_cd sh_phq4 if demo_age==3
svy:regress sas time_sns_cati  i.demo_sex i.demo_edu   i.demo_income i.demo_maritalstatus i.demo_employment
i.time_internet_cati i.time_book_cati i.time_video_cati i.sh_smoking i.sh_drinking i.sh_cd sh_phq4 if demo_age==4

svy:regress sas time_sns_cati  i.demo_age i.demo_sex  i.demo_income i.demo_maritalstatus i.demo_employment
i.time_internet_cati i.time_book_cati i.time_video_cati i.sh_smoking i.sh_drinking i.sh_cd sh_phq4 if demo_edu==1
svy:regress sas time_sns_cati  i.demo_age i.demo_sex  i.demo_income i.demo_maritalstatus i.demo_employment
i.time_internet_cati i.time_book_cati i.time_video_cati i.sh_smoking i.sh_drinking i.sh_cd sh_phq4 if demo_edu==2
svy:regress sas time_sns_cati i.demo_age i.demo_sex  i.demo_income i.demo_maritalstatus i.demo_employment
i.time_internet_cati i.time_book_cati i.time_video_cati i.sh_smoking i.sh_drinking i.sh_cd sh_phq4 if demo_edu==3

svy:regress sas time_sns_cati i.demo_age i.demo_sex i.demo_edu i.demo_maritalstatus i.demo_employment
i.time_internet_cati i.time_book_cati i.time_video_cati i.sh_smoking i.sh_drinking i.sh_cd sh_phq4 if
demo_income_cati ==1
svy:regress sas time_sns_cati i.demo_age i.demo_sex i.demo_edu i.demo_maritalstatus i.demo_employment
i.time_internet_cati i.time_book_cati i.time_video_cati i.sh_smoking i.sh_drinking i.sh_cd sh_phq4 if
demo_income_cati ==2
```

```
svy:regress sas time_sns_cati  i.demo_age i.demo_sex i.demo_edu i.demo_maritalstatus i.demo_employment
i.time_internet_cati i.time_book_cati i.time_video_cati i.sh_smoking i.sh_drinking i.sh_cd sh_phq4 if
demo_income_cati ==3
```
